# Conjugation of Synthetic Polyproline Moietes to Lipid II Binding Fragments of Nisin Yields Active and Stable Antimicrobials

**DOI:** 10.3389/fmicb.2020.575334

**Published:** 2020-11-20

**Authors:** Jingjing Deng, Jakob H. Viel, Vladimir Kubyshkin, Nediljko Budisa, Oscar P. Kuipers

**Affiliations:** ^1^Department of Molecular Genetics, University of Groningen, Groningen, Netherlands; ^2^Institute of Chemistry, Technical University of Berlin, Berlin, Germany; ^3^Department of Chemistry, University of Manitoba, Winnipeg, MB, Canada

**Keywords:** click chemistry, RiPPs, lantibiotics, nisin, polyproline peptides

## Abstract

Coupling functional moieties to lantibiotics offers exciting opportunities to produce novel derivatives with desirable properties enabling new functions and applications. Here, five different synthetic hydrophobic polyproline peptides were conjugated to either nisin AB (the first two rings of nisin) or nisin ABC (the first three rings of nisin) by using click chemistry. The antimicrobial activity of nisin ABC + O6K3 against *Enterococcus faecium* decreased 8-fold compared to full-length nisin, but its activity was 16-fold better than nisin ABC, suggesting that modifying nisin ABC is a promising strategy to generate semi-synthetic nisin hybrids. In addition, the resulting nisin hybrids are not prone to degradation at the C-terminus, which has been observed for nisin as it can be degraded by nisinase or other proteolytic enzymes. This methodology allows for getting more insight into the possibility of creating semi-synthetic nisin hybrids that maintain antimicrobial activity, in particular when synthetic and non-proteinaceous moieties are used. The success of this approach in creating viable nisin hybrids encourages further exploring the use of different modules, e.g., glycans, lipids, active peptide moieties, and other antimicrobial moieties.

## Introduction

Nisin is the first discovered and the best studied lantibiotic and it is produced by *Lactococcus lactis* (Rogers, 1928). In addition to its natural presence in fermented foods, nisin has been applied as a food preservative for many decades, because of its excellent activity against food spoilage (Hansen and Sandine, 1994; Gharsallaoui et al., 2016). Beyond its role in food safety and preservation, nisin has potential therapeutic applications. It is for instance effective against many Gram-positive antibiotic-resistant organisms, such as methicillin-resistant *Staphylococcus aureus* (MRSA) and vancomycin-resistant *Enterococcus* (VRE) (Shin et al., 2016). The exceptional activity of nisin is derived from a unique structure, containing one lanthionine and four methyllanthionine rings, which has a dual mode of action. The first two rings (AB) form a lipid II recognition site. By binding to the peptidoglycan precursor lipid II, nisin inhibits cell wall biosynthesis. The last two rings (DE), which are connected to rings ABC through a hinge region, constitute a membrane insertion domain. After rings AB dock to lipid II (Brötz et al., 1998), rings DE and the tail can insert into the bacterial membrane to create pores, where nisin forms pores (Lubelski et al., 2008). Nisin’s dual mode of action increases its antimicrobial activity, and decreases the chance of resistance development in target organisms. These features make nisin an attractive candidate for development into an antibiotic alternative. Unfortunately, nisin is readily degraded in the gut, which precludes oral delivery. Also, the administering of nisin by injection, especially at its full-length, is limited by the instability of its dehydro-residues. If possible, these problems should be addressed to broaden the scope of nisin application in the therapeutic setting. A promising strategy to achieve this has been provided by the chemical coupling of specific (protease resistant) moieties to nisin, and semi-synthetic fragments of nisin. This approach allows for the synthesis of novel derivatives with useful properties like increased stability, alleviating some of nisin’s characteristics that are problematic in its potential role as an antibiotic alternative.

The coupling of peptides to compatible moieties can be achieved through the widely used click chemistry method (Ahmad Fuaad et al., 2013). “Copper (I)-catalyzed azide-alkyne cycloaddition (CuAAC)” is the most common reaction representing the click chemistry. It is a region selective copper (I) catalytic cycloaddition reaction between an azide and an alkyne leading to the formation of a triazole. The molecules connected to the respective reagents are effectively “clicked” together (Kolb et al., 2001). Due to its reliability, specificity, biocompatibility, easiness to perform, and mild reaction conditions, click chemistry is being used increasingly in diverse areas, such as bioconjugation, drug design and polymer science (Thirumurugan et al., 2013; McKay and Finn, 2014; Jiang et al., 2019). The success of click chemistry in the field of peptide modification can be attributed to the resulting triazole ring which resembles an amide bond, and which increases the stability of the resulting molecule. This is achieved at least in part by increasing the molecule’s resistance to proteases, as the triazole aligns with the biological targets through hydrogen bonding and dipole interactions (Ahmad Fuaad et al., 2013). Peptide coupling through click chemistry has been the subject of several studies toward the development of target-specific bacterial probes and expanding application possibilities of this method (Arnusch, 2008; Yoganathan et al., 2011; Oldach et al., 2012; Slootweg et al., 2013a, 2014; Koopmans et al., 2015; Bolt et al., 2018). A prominent example of applying click chemistry to enhance lantibiotics, is that of coupling nisin AB to lipid moieties (Koopmans et al., 2015). The resulting hybrid molecules exhibited increased stability, as well as potent antimicrobial activity against drug-susceptible and -resistant strains of Gram-positive bacteria. In other studies, the lipid II-binding motif (rings AB) of nisin has been conjugated with various functional molecules (Arnusch, 2008; Koopmans et al., 2015; Bolt et al., 2018).

**FIGURE 1:**
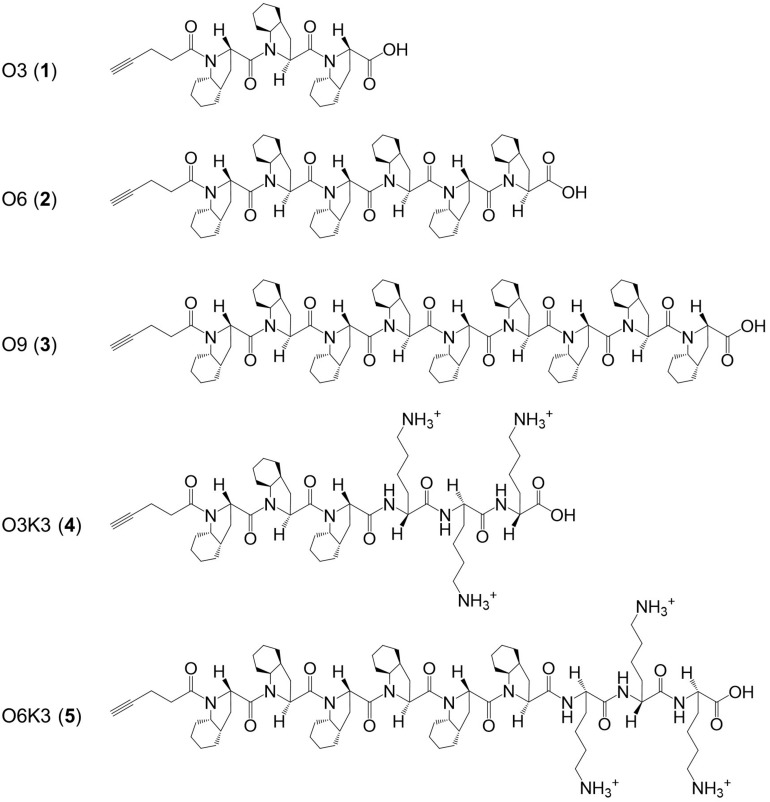
Structure of the hydrophobic polyproline peptides O3, O6, O9, O3K3, and O6K3 for putative membrane anchoring. The peptides were constructed with a hydrophobic proline analog (*2S,3aS,7aS*)-octahydroindole-2-carboxylic acid (Oic, designated as O) and lysine residues (Lys, K).

Here, a range of experiments was designed for the synthesis of nisin hybrids by coupling specific synthetic polyproline peptides, some of which containing cationic residues, to either nisin AB or nisin ABC. These polyproline peptides (Figure 1) were designed based on a polyproline structural skeleton using a proline analog [(*2S,3aS,7aS*)-octahydroindole-2-carboxylic acid, Oic] to display a linear and hydrophobic structure affine to a lipid membrane (Kubyshkin and Budisa, 2018; Kubyshkin et al., 2019). The selected properties of these nisin hybrids should aid in the membrane translocation of their C-terminal region and, as the molecular weight of the clicked polyproline moieties ranges between 0.5 and 1.5 kDa, the resulting molecules remain well under nisin in size. Hypothetically, the lipid-II targeting nisin AB would guide the conjugate to the bacterial membrane, where the hydrophobic tail would flip into the membrane core, tightly anchoring the conjugate. The newly synthesized nisin hybrids were compared to nisin with regard to their antimicrobial activity, and susceptibility to proteolytic degradation.

## Materials and Methods

### Bacterial Strains and Plasmids

Indicator strains and plasmids used in this work are given in Table 1.

**TABLE 1:** Strains and plasmids used in this work.

Indicator strains	Characteristics	References
*Micrococcus flavus*		Lab collection
*Lactococcus lactis* NZ9000		Lab collection
*Staphylococcus aureus* MW2	Methicillin resistant (MRSA)	The University Medical Center Groningen, The Netherlands
*Enterococcus faecium* LMG 16003	Avaparicin and vancomycin resistant (VRE)	Laboratory of Microbiology, Gent, Belgium
*Listeria monocytogenes* LMG 10470		Montalbán-López et al., 2018
**Plamids**	**Characteristics**	**References**
pEmpty	pNZ8048, pSH71 origin of replication, P*_*n*__*isA*_* promoter and empty multiple cloning site, chloramphenicol resistance	Kuipers et al., 1998
pNSR	pNZ-SV-SaNSR, pSH71 origin of replication, expression of nsr under the control of P*_*n*__*isA*_* promoter, chloramphenicol resistance	Khosa et al., 2013, 2016

### Preparation of Nisin AB-Azide

Nisin AB was purified in accordance with protocols reported previously (Slootweg et al., 2013b). Briefly, nisin (180 mg) was dissolved in 150 mL Tris buffer (5 mmol Tris acetate, 5 mmol CaCl_2_, 25 mmol sodium acetate, pH 7.0) and the solution was cooled on ice for 15 min. Then trypsin (15 mg) was added and warmed up to room temperature for 15 min. The reaction was performed at 30°C for 16 h and an extra 15 mg trypsin was added. After 24 h incubation, another 15 mg trypsin was added and the reaction was performed for another 24 h. The reaction mixture was acidified with hydrochloric acid (1 M) to pH 4.0 followed by adding 3 mL acetonitrile and concentrated *in vacuo*. The pure nisin AB was purified from the mixture by RP-HPLC with the water-acetonitrile gradient mobile phase containing trifluoroacetic acid (0.1%) and lyophilized to obtain a white powder (20 mg). Nisin AB (10 mg, 8.6 μmol) was dissolved in *N,N*-dimethylformamide (DMF) (100 μl) and azidopropylamine (44 μl, 43.2 mg, 432 μmol), *N,N*-diisopropylethylamine (DIPEA, 6 μl, 34.8 μmol), and (benzotriazol-1-yloxy)-tris-(dimethylamino) phosphonium hexafluorophosphate (BOP, 7.6 mg, 17.2 μmol) or (benzotriazol-1-yl-oxy)-tris-(pyrrolidino)phosphonium hexafluorophosphate (PyBOP, 9 mg, 17.2 μmol) were added. The reaction was vortexed for 20 min and subsequently quenched with 5 mL buffer (water : acetonitrile, 95:5 vol+ 0.1% trifluoroacetic acid). The reaction mixture was purified by RP-HPLC with the water-acetonitrile gradient mobile phase containing trifluoroacetic acid (0.1%) and pure nisin AB-azide was lyophilized to obtain a white powder (8 mg).

### Preparation of Nisin ABC-Azide

α-chymotrypsin was used to digest nisin to generate nisin ABC. Nisin (180 mg) was dissolved in 150 mL Tris buffer (25 mmol Tris acetate, pH7.5) and the solution was cooled on ice for 15 min. Then α-chymotrypsin (15 mg) was added and warmed up to room temperature for 15 min. The enzymatic digestion was performed same as described for nisin AB. Nisin ABC was purified from the mixture by RP-HPLC with the water-acetonitrile gradient mobile phase containing trifluoroacetic acid (0.1%) and then lyophilized to obtain a white powder (20 mg). Nisin ABC (10 mg, 6.5 μmol) was dissolved in DMF (50 μl). The azide-coupling reaction was performed same as described for nisin AB.

### Preparation of the Hydrophobic Polyproline Peptides

The polyproline-containing peptides were prepared using a manual Fmoc-based solid-phase peptide synthesis scheme as described (Kubyshkin and Budisa, 2018). The sequences were grown on 2-clorotrityl resins pre-loaded with either Fmoc-Oic-OH or Fmoc-Lys (Boc)-OH (Fmoc = fluorenylmethyloxycarbonyl, Boc = tert-butyloxycarbonyl). The resin loading was estimated at 0.7–0.8 mmol/g. The synthesis was performed in DMF using 2.5 equiv. of the Fmoc-amino acid pre-activated with the mixture of 2.5 equiv. 2-(1H-benzotriazole-1-yl)-1,1,3,3-tetramethylaminium tetrafluoroborate (TBTU) and 2.5 equiv.1-hydroxybenzotriazole (HOBt) mixture under addition of 5 equiv. DIPEA. The N-terminal pentynyl moiety was installed under coupling with pentynoic acid under same activation conditions. The Fmoc group was removed by treatment with 22 vol% piperidine in DMF. The final peptides were cleaved off the resin by treatment with hexafluoroisopropanol : dichloromethane (1:3, vol: vol) mixture. The peptides were additionally purified on short silica gel columns using dichloromethane-methanol gradient elution. Pentynyl-(Oic)9-OH (O9) peptide was additionally purified by precipitation from methanol. The Boc-group was removed from the lysine side-chains by treatment with 4 M hydrogen chloride in dioxane. The identity and purity of the final peptides were confirmed by mass-spectra (ESI-Orbitrap) and ^1^H-NMR spectra (CD_3_OD, 700 MHz). The peptides were obtained in 10–50 mg quantities.

### Click Chemistry

**FIGURE 2:**
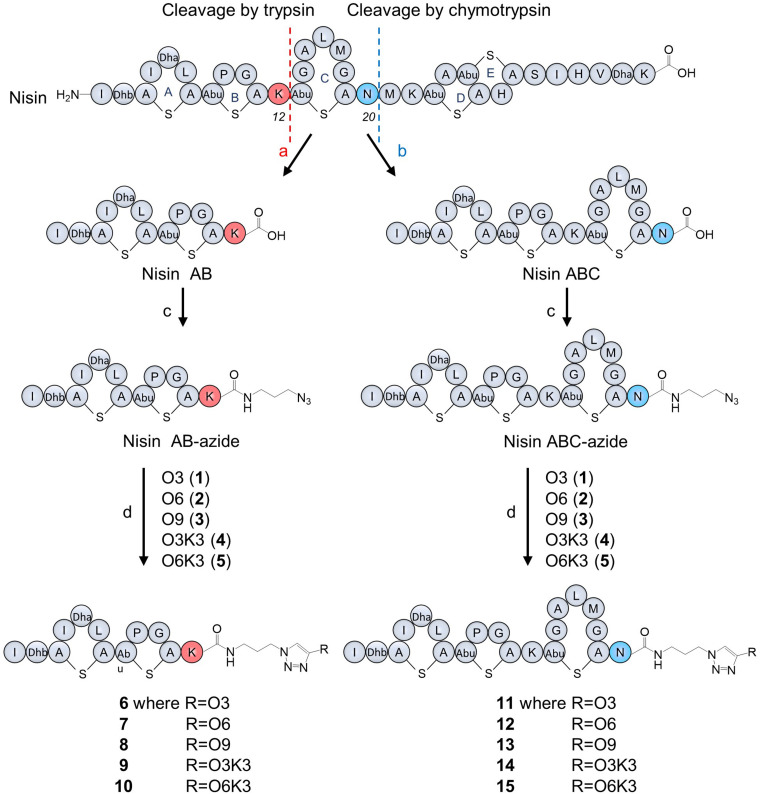
Nisin digestion and semi-synthesis of nisin AB and nisin ABC conjugates. **(a)** Trypsin, Tris buffer, pH 7.0, 30°C, 48 h; **(b)** chymotrypsin, Tris buffer, pH 7.5, 30°C, 48 h; **(c)** azidopropylamine, PyBOP, DIPEA, DMF, RT, 20 min; **(d)** CuSO_4_, BTTAA, sodium ascorbate in phosphate buffer, 37°C, 1 h.

A stock solution of CuSO_4_ (10 mg, 100 mM), sodium ascorbate (200 mg, 1 M) 2-(4-((bis((1-(tert-butyl)-1H-1,2,3-triazol-4-yl)methyl)amino)methyl)-1H-1,2,3-triazol-1-yl)-acetic acid (BTTAA, 25 mg, 50 mM), and tris((1-hydroxy-propyl-1H-1,2,3-triazol-4-yl)methyl)amine (THPTA, 25 mg, 250 mM) in deionized water and a stock solution of O3 (1 mg, 36 mM), O6 (1.8 mg, 36 mM), O9 (2.6 mg, 36 mM), O3K3 (1.9 mg, 36 mM) and O6K3 (2.7 mg, 36 mM) in DMF (50 μl) were prepared, aliquoted and stored at −20°C for further use. Firstly, nisin AB-azide (25 μg, 0.02 μmol) or nisin ABC-azide (40 μg, 0.02 μmol) were dissolved in 100 mM phosphate buffer (pH7.0, final reaction volume: 200 μl). Then, the appropriate O3, O6, O9, O3K3, or O6K3 (5 μl, 0.18 μmol) and CuSO_4_ (4 μl, 0.4 μmol) : THPTA (8 μl, 2 μmol) or BTTAA (40 μl, 2 μmol)-premix were added followed by the addition of sodium ascorbate (20 μl, 20 μmol). The reaction was performed at 37°C for 1 h and purified directly by RP-HPLC with the water-acetonitrile gradient mobile phase containing trifluoroacetic acid (0.1%). The pure product-containing fractions were lyophilized to obtain nisin hybrids **6**–**15** as white fluffy powders (Figure 2). The reaction was further scaled up in ratio to obtain more products.

### Agar Well Diffusion Assay

Agar well diffusion assay against *Micrococcus flavus* was performed as described previously (van Heel et al., 2013). 0.15 nmol of each sample was added to each well. The agar plate was incubated at 30°C overnight, after which the zone of inhibition was measured.

### Determination of the Minimal Inhibitory Concentration (MIC)

All samples were resuspended in 0.05% aqueous acetic acid solution and the peptide amount was quantified by HPLC according to Schmitt et al. (Schmitt et al., 2019). The indicator strains MW2-MRSA, *Enterococcus faecium, Listeria monocytogenes*, *and Lactococcus lactis* were first streaked on GM17 plate and cultured overnight. The peptide samples were diluted with 0.05% acetic acid to a concentration of 40–320 μM (depending on the estimated activity of the peptide and the strain tested). The MIC value test was performed according to Wiegand et al. (2008).

## Results

### Production of Nisin AB-Azide and Nisin ABC-Azide

Nisin was digested using trypsin and chymotrypsin, respectively, to generate nisin AB and nisin ABC fragments (Figure 2). The desired truncated nisin molecules were purified from the digestion mixture with yields in the milligram range, in accordance with protocols reported previously (Slootweg et al., 2013b). After purification, an azide linker was added to the C-terminus of the acquired nisin fragments. Since the truncated variants with the azide linker were needed in relatively large quantities for the generation of the semi-synthetic analogs, the previously reported peptide coupling procedure was optimized for this study. Initially, addition of the azide linker was done by coupling azidopropylamine to nisin AB in the presence of BOP as the coupling reagent. HPLC analysis of the reactions performed under these conditions showed substantial amounts of substrate remained unreacted, resulting in a reaction efficiency of only 7.4% (Supplementary Figure 1). Prolonging the reaction time to 1 h did not increase the conversion. However, by substituting the coupling reagent BOP for PyBOP, the reaction efficiency could be increased to 89% (Supplementary Figure 1). For reactions of this nature, PyBOP was shown to be a better coupling reagent than to BOP. Using the optimized conditions from the above experiment, azidopropylamine was coupled to nisin AB and nisin ABC in a reaction containing PyBOP/DIPEA to give nisin AB-azide and nisin ABC-azide in 89 and 87% yield, respectively, which was purified by HPLC and characterized by MALDI-TOF. The resulting nisin AB-azide and nisin ABC-azide contain convenient handles for ligation to alkynes via CuAAC.

### Production of Nisin AB and Nisin ABC Conjugates

Five hydrophobic polyproline peptides (**1**–**5**) were coupled to nisin AB-azide and nisin ABC-azide (Figure 2). The first click reaction was attempted with O3 (**1**) and nisin AB-azide in the presence of THPTA as copper (I)-stabilizing ligand. Analysis by HPLC showed that a good amount of product was formed under these reaction conditions. Increasing the reaction temperature to 50°C and extending the reaction time to 2 h led to degradation rather than increased conversion. The conversion could however be increased by using BTTAA as substitute for THPTA as copper (I)-stabilizing ligand improved the conversion. The best results were obtained using 9 equiv. O3, 20 equiv. CuSO_4_, 100 equiv. BTTAA and 1,000 equiv. sodium ascorbate, reacted at 37°C for 1 h. Under these optimized conditions, the click reaction of nisin AB-azide and nisin ABC-azide with the five hydrophobic polyproline peptides (**1**–**5**) were carried out successfully to give semi-synthetic nisin hybrids **6**–**15** in 42–54% yields. The resulting semi-synthetic nisin hybrids **6**–**15** were further characterized by MALDI-TOF.

### Antimicrobial Activity of Nisin AB and Nisin ABC Conjugates

**FIGURE 3:**
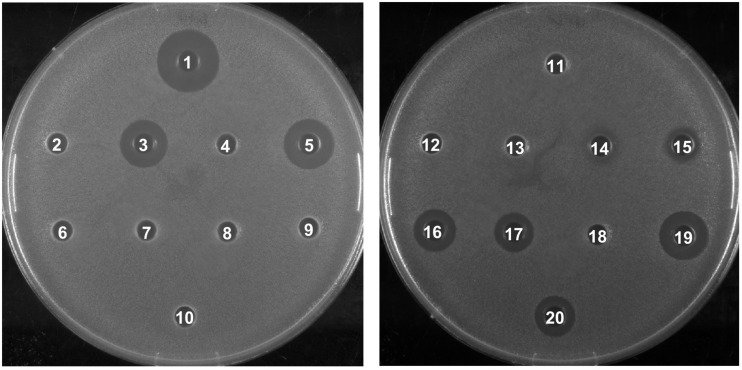
Antimicrobial activity of semi-synthetic nisin hybrids against *M. flavus* by agar well diffusion assay. 1: Nisin; 2: Nisin AB; 3: Nisin ABC; 4: Nisin AB-azide; 5: Nisin ABC-azide; 6: O3; 7: O6; 8: O9; 9: O3K3; 10: O6K3; 11: Nisin AB + O3; 12: Nisin AB + O6; 13: Nisin AB + O9; 14: Nisin AB + O3K3; 15: Nisin AB + O6K3; 16: Nisin ABC + O3; 17: Nisin ABC + O6; 18: Nisin ABC + O9; 19: Nisin ABC + O3K3; 20: Nisin ABC + O6K3.

To investigate the biological activity of the nisin hybrids, an agar well diffusion assay and a growth inhibition assay were performed. *M. flavus* was used as the indicator strain for the agar well diffusion assay, and 0.15 nmol of each sample was added to each well (Figure 3). The results showed that nisin AB and five hydrophobic polyproline moieties (**1**–**5**) are not active alone and nisin has the highest activity. Of the nisin AB conjugates, nisin AB + O6K3 is the only active one. Notably, with the exception of nisin ABC + O9, all four nisin ABC conjugates showed activity. Most notably the activity of nisin ABC + O3K3 is considerably higher than that of nisin ABC. Antimicrobial activity of all compounds was tested by growth inhibition assays against two clinically relevant Gram-positive pathogens, i.e., methicillin resistant *S. aureus* and vancomycin resistant *E. faecium*, as well as *L. monocytogenes*, and *L. lactis*. Their minimal inhibitory concentration (MIC) was determined using an established broth microdilution assay (Table 2), using nisin as a positive control. Nisin AB was devoid of activity at the highest concentration tested except against *E. faecium*. Since of the nisin AB conjugates only nisin AB + O6K3 showed activity in the agar well diffusion assay, they were only tested against *L. lactis*. In this growth inhibition assay, nisin AB + O9 showed the best activity among the five nisin AB conjugates. Nisin ABC conjugates displayed a retained or even increased activity against *E. faecium* and *L. lactis* compared to nisin ABC alone, whereas activity against MW2-MRSA diminished. The antimicrobial activity of nisin ABC + O6K3 against *E. faecium*, *L. monocytogenes*, and *L. lactis* decreased only 8-, 4-, and 12-fold compared to full nisin, respectively. Strikingly, its antimicrobial activity against *E. faecium*, *L. monocytogenes*, and *L. lactis* increased 16-, 4-, and 2-fold compared to nisin ABC, respectively, and increased all twice compared to nisin ABC + O6, respectively. Compared to nisin ABC, nisin ABC + O6K3 displayed improved activity against *E. faecium*, *L. monocytogenes*, and *L. lactis* but decreased activity against MW2-MRSA while nisin ABC + O9 showed enhanced activity against *E. faecium* although activity against other strains was retained or even reduced. An additional test was performed to assess if the resistance to proteolytic degradation of the semi-synthetic nisin hybrids had improved compared to the parent compound. Nisin, nisin ABC + O6K3, and nisin ABC + O6 were exposed to the nisin resistance protein (NSR), a peptidase that cleaves the linear C-terminus of nisin. For this experiment, an activity test was performed against the NSR producing strain *L. lactis* NZ9000 (pNSR). In this test, NSR conferred its producing strain over 16-fold resistance toward nisin in MIC, caused by the proteolytic cleavage at the C-terminal tail of nisin. The hybrids Nisin ABC + O6K3 and nisin ABC + O6 bypassed this nisin resistance mechanism, having identical MICs against NZ9000 regardless of it producing NSR.

**TABLE 2:**
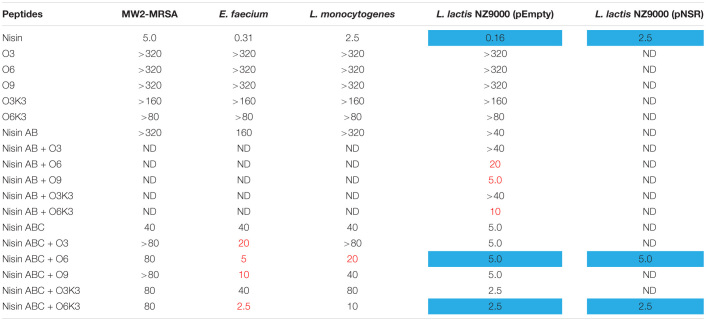
MIC value (μM) of nisin AB and nisin ABC conjugates.

**In red: MIC values that are improved in comparison to one of nisinAB or nisin ABC. In blue: the effect of the nisinase presence is depicted. ND: not determined.**

## Discussion

In this research, an efficient and direct method for the preparation of nisin hybrids was developed. Nisin AB and nisin ABC fragments were obtained by enzymatic digestion of nisin and these fragments were subsequently C-terminally functionalized with azidopropylamine. Five hydrophobic polyproline peptides (without and with cationic residues) were synthesized and coupled to nisin AB-azide and nisin ABC-azide by using click chemistry. Ten newly synthesized nisin hybrids were obtained and their antimicrobial activities were tested. The agar diffusion assay showed that the activity of nisin ABC conjugates are much better than nisin AB conjugates. These results are in line with previous studies that showed that variants lacking ring C, or where ring C is not closed, lack antimicrobial activity (Chan et al., 1996). It is noteworthy that while nisin AB is inactive having a lysine at the C-terminus, it gains higher activity through conjugation with O6K3 than with the more hydrophobic O6. The growth inhibition experiments showed that the activity of nisin ABC + O6K3 are better than nisin ABC + O6 and nisin ABC + O9, again indicating that addition of lysines (positive charge) at the C-terminal region can improve the activity. The antimicrobial activity of nisin ABC + O6K3 against *E. faecium* was 8-fold less active than full-length nisin. However, the activity was 16-fold better than nisin ABC, suggesting that modifying nisin ABC is a promising strategy to generate semi-synthetic nisin hybrids. It is notable that the inhibition activities of the semi-synthetic hybrids did not fully correlate when comparing solid media tests and broth MIC tests. However, this effect has been described for many nisin mutants, e.g., nisin A and nisin Z (de Vos et al., 1993). These compounds have an identical MIC, but a single amino acid difference leads to different halo sizes on diffusion assays, which is likely caused by altered diffusion properties. The five polyproline moieties that were tested in this study have varying hydrophobicities. Therefore, the lack of correlation between both essay results are likely caused by their distinct diffusion characteristics. In addition to their increased activity, these variants are not prone to degradation at the C-terminus by NSR, as was observed for nisin. Although the full proteolytic resistance of the conjugates was not tested, polyproline chains commonly have resistance against proteolysis in general, and convey this property to the nisin hybrids, providing a proof of concept. Notably, the method described in this study can be applied to conjugate other compatible (synthetic and non-proteinaceous) moieties that can provide the desired resistance to specific proteases. Nisin AB-azide and nisin ABC-azide can be readily generated with yields in the milligram range according to our optimized protocol. Future studies may focus on coupling peptides, especially anti-Gram-negative peptides, with nisin ABC-azide. Overall, this study highlights how lantibiotic fragments can be used as lead structures to create novel variants with altered properties (e.g., stability, activity, and specificity) via chemical coupling.

## Data Availability Statement

All datasets generated for this study are included in the article/Supplementary Material, further inquiries can be directed to the corresponding author.

## Author Contributions

JD and OK planned, conceived, and analyzed the experiments. VK designed the polyproline moieties. JD, JV, and VK performed the experiments. JD, VK, NB, and OK drafted the manuscript and contributed to the data interpretation. All authors read, critically revised, and approved the final manuscript.

## Conflict of Interest

The authors declare that the research was conducted in the absence of any commercial or financial relationships that could be construed as a potential conflict of interest.
