# Supplementary material for: Conjugation of Synthetic Polyproline Moietes to Lipid II Binding Fragments of Nisin Yields Active and Stable Antimicrobials
Source: Front Microbiol. 2020 Nov 20;11:575334. doi: 10.3389/fmicb.2020.575334 (PMC7715017; doi:10.3389/fmicb.2020.575334)
Supplement: Supplementary file 1 [file Presentation_1.pdf]

Supplementary Material

1. Suplementary Figure

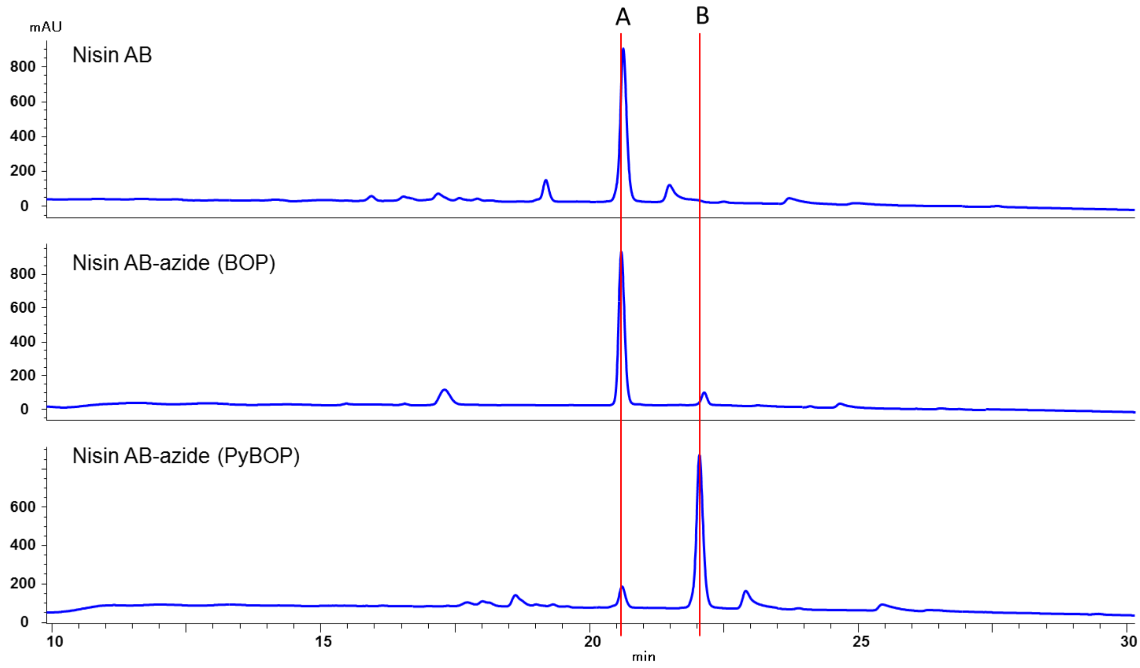

Supplementary Figure 1: HPLC analysis of reaction substrate nisin AB and the reaction mixtures using BOP and PyBOP. A: peak of substrate; B: peak of product. Conditions: RP 18 column 4.6 × 250 mm, solvent A, H_2O with 0.1% TFA; solvent B, acetonitrile with 0.1% TFA; flow rate 1 mL/min for a 45 min run. A gradient (0-5 min 20% solvent B, 5-30 min linear to 60% solvent B; 30-31 min linear to 100% solvent B and within 45 min back to 20% solvent B) was used.
